# Medical Society of the County of Erie

**Published:** 1902-08

**Authors:** Franklin C. Gram


### SOCIETY PROCEEDINGS.

### Medical Society of the County of Erie.

*Semi-Annual Meeting.*

Reported by FRANKLIN C. GRAM, M. D., Secretary.

THE semi-annual meeting of the Medical Society of the County of Erie was held in the Y. M. C. A. parlors, Pearl and Mohawk Streets, Buffalo, June 17, 1902.

The president, Dr. W. M. Ward, called the meeting to order at 10.30 a.m., and the secretary read the minutes of the previous meeting which were approved.

Dr. William Warren Potter, chairman of the membership committee, presented the names of Dr. Joseph Burke, Dr. Adolph H. Urban and Dr. H. L. Atwood, and recommended their election as members. The recommendation was adopted. Dr. Potter also reported for the committee on legislation that the usual attempts to pass injurious medical laws were made at the last session of the state legislature, but through the combined efforts of various committees they had failed.

Dr. J. B. Coakley, chairman of the Board of Censors, submitted the following report, which was adopted:

*To the Medical Society of the County of Erie:*

Your board of censors respectfully reports that since its last report made to the society, January, 1902, the censors have continued their usual methods of work in the investigation of cases of alleged illegal practitioners of medicine and surgery.

The most important matter which was pending at the date of our last report was the action against "Professor" A. J. Wiechers, otherwise known as "Antonius, the Boy Phenomenon." At the date of our last report Wiechers was under bail to appear before the Grand Jury and answer to the charge of the illegal practice of medicine. Since that time, his case has been presented to the Grand Jury which, acting under the advice of the District Attorney's office, refused to find an indictment. The chairman of the board of censors had quite an extended interview with the staff of the District Attorney's office in reference to this case and was informed that the District Attorney's office had advised the Grand Jury not to bring in an indictment against Prof. Wiechers for the reason that it could not be shown that Prof. Wiechers had personally ever received any monies or compensation for his treatment from his patients, but that all monies paid for such treatments had been received by others, in most cases duly licensed physicians, who were so lacking in principle and regard for the standing and dignity of their profession that they had permitted themselves to be employed by this alleged healer as members of his staff to further his unworthy ends. Furthermore, the District Attorney's office insists that the massage treatment, manipulation, and rubbing, practised by the Boy Phenomenon does not come under the head or within the definition of the practice of medicine.

The District Attorney and his assistants seemed to take the position that they are the judges of what constitutes the practice of medicine rather than the members of the medical profession, and by this prejudging the case refused to permit these alleged violations of the law to be submitted to a jury in the light of the facts and the interpretation to be placed upon medical terms and the meaning of the phrase "Practice of Medicine" by members learned in that profession. The District Attorney's office although differing from your board as to what constitutes illegal practice of medicine has at all times been very courteous in its treatment of your board of censors and has shown every disposition to assist the board in every wây possible, according to their understanding of the legal situation, in the prevention of the violation of the law and punishment of those guilty of such violation.

Since our last semi-annual report your board has also carefully prepared a list of the names of persons given in the Buffalo directory as physicians, who were not duly registered as such and a circular letter was sent to each of said persons, about thirty in all. Of this number to whom such circular letter was addressed seven were unable to be found in the city by the post office authorities and the letters were returned with the statement that the several parties had left the city. Two more have left the city since. Of the remainder, seven were found to be dentists, one a doctor of philosophy, one a plumber, and one a medical student, whose name through mistake or inadvertence had been inserted in the list of practising physicians. However, as a result of the efforts of your board in this direction at least nine persons who were presumably illegally practising medicine in the city have been forced to leave town.

Since our last semi-annual report several cases of chiropodists and magnetic healers illegally practising medicine or using the title of "Dr." have been reported to your board and investigated, and in nearly every instance persons against whom such charges have been brought have either left the city or discontinued such illegal practice.

All of which is respectfully submitted.

J. B. Coakley, Chairman.
H. R. Hopkins,
Irving W. Potter,
Francis E. Fronczak,
Henry Lapp.

Dated Buffalo, N. Y., June 17, 1902.

A donation of $5.00 was voted the janitor of the building for preparing the rooms, as no rental is demanded by the Association for their use.

The members then listened to a very instructive and interesting paper on

NASAL DEFORMITIES AND THEIR CORRECTION,

By Dr. JOHN O. ROE, of Rochester. Various illustrations and photographs were shown, and materially assisted in demonstrating a vivid description of cases by the lecturer. A cordial vote of thanks was tendered Dr. Roe for his able address.

Dr. WILLIAM C. KRAUSS gave a history and description of a case of aortic aneurism and presented the subject.

Dr. JOSEPH BURKE read an exhaustive paper on

CONGENITAL PULMONARY STENOSIS (See page 15).

Dr. WILLIAM WARREN POTTER, president of the State Medical Examining Board, gave a brief rèsumè of the various efforts and events leading to the enactment of a state law regulating the practice of medicine, and the work thus far accomplished under this law. Dr. Henry R. Hopkins, president of the Medical Society of the State of New York, also discussed the subject. All the lecturers received a hearty vote of thanks.

